# Longitudinal correlation between X‐ray and MRI findings in medial compartment knee osteoarthritis: Insights into early cartilage loss and structural changes

**DOI:** 10.1002/ksa.70016

**Published:** 2025-08-31

**Authors:** Do Weon Lee, Ji‐Sahn Kim, Hyuk‐Soo Han, Du Hyun Ro

**Affiliations:** ^1^ Department of Orthopedic Surgery Dongguk University Ilsan Hospital Goyang South Korea; ^2^ Department of Orthopedics Seoul National University College of Medicine Seoul South Korea; ^3^ Department of Orthopedic Surgery Seoul National University Hospital Seoul South Korea; ^4^ Healthcare AI Research Institute Seoul National University Hospital Seoul South Korea; ^5^ CONNECTEVE Co., Ltd Seoul South Korea

**Keywords:** cartilage, magnetic resonance imaging, knee, osteoarthritis, radiography

## Abstract

**Purpose:**

Knee osteoarthritis (OA) is a common joint disorder assessed using radiographic (X‐ray) and magnetic resonance imaging (MRI). While X‐rays are accessible, MRI provides detailed insights into meniscus and cartilage. Few studies have evaluated the correlation between X‐ray and MRI findings in knee OA longitudinally. This study addresses this gap by investigating their relationship over time.

**Methods:**

The Multicenter Osteoarthritis Study (MOST) dataset, a public, longitudinal cohort study focusing on knee OA in older adults, was used. The analysis included 3710 knees with medial compartment OA from different follow‐ups over the course of 7 years. X‐ray findings were compared with MRI findings, encompassing cartilage, meniscal and bone pathologies.

**Results:**

In the central compartment, knee OA progression began with cartilage loss, followed by meniscal and bone pathology, while in the posterior compartment, meniscal changes preceded cartilage and bone lesions. Cartilage loss in the central femur was the earliest event, even in Kellgren–Lawrence grade 0 knees, preceding X‐ray changes. Tibial osteophytes developed first on X‐ray, followed by joint space narrowing and femoral osteophytes. Longitudinal regression identified meniscal extrusion, cartilage loss and meniscal tear as significant predictors of OA progression (*p* < 0.001), with meniscal extrusion being the strongest.

**Conclusion:**

Knee OA progression differs by compartment, with cartilage loss initiating changes centrally and meniscal pathology leading posteriorly. Tibial osteophytes appear early on X‐ray. Meniscal extrusion is the strongest predictor of OA progression, highlighting the importance of MRI in identifying early changes and guiding personalised management.

**Level of Evidence:**

Level II.

**Abbreviations**
BMFMC: centro‐medial femur bone marrow lesion
BMFMP: postero‐medial femur bone marrow lesion
BMI: body mass index
BMTMC: centro‐medial tibia bone marrow lesion
BMTMP: postero‐medial tibia bone marrow lesion
CMFMC: centro‐medial femur cartilage morphology
CMFMP: postero‐medial femur cartilage morphology
CMTMC: centro‐medial tibia cartilage morphology
CMTMP: postero‐medial tibia cartilage morphology
JSN: joint space narrowing
KL: Kellgren–Lawrence
MOST: Multicenter Osteoarthritis Study
MRI: magnetic resonance imaging
MTMB: medial body meniscal tear
MTMP: postero‐medial meniscal tear
OA: osteoarthritis
OARSI: Osteoarthritis Research Society International
WORMS: whole‐organ magnetic resonance imaging score
XRJSL: lateral joint space narrowing grade
XRJSM: medial joint space narrowing grade
XROSFL: lateral femoral osteophyte grade
XROSFM: medial femoral osteophyte grade
XROSTL: lateral tibial osteophyte grade
XROSTM: medial tibial osteophyte grade

## INTRODUCTION

Knee osteoarthritis (OA) is a prevalent and disabling joint disorder that affects millions worldwide, particularly among older adults. The disease is characterised by progressive cartilage degradation, joint space narrowing, osteophyte formation and ultimately, loss of joint function and mobility. Knee OA is one of the leading causes of pain and disability in older populations, contributing to significant healthcare costs and societal burden [16]. Early detection of OA is critical as it offers the best opportunity for interventions that may slow disease progression, alleviate symptoms and enhance quality of life [7]. However, early‐stage OA often goes undetected due to the insidious nature of its structural changes, making timely and accurate diagnostic tools essential [18].

Radiography (X‐ray) and magnetic resonance imaging (MRI) are the primary imaging modalities used to assess OA‐related structural changes. X‐ray is widely accessible, relatively low‐cost and frequently used in clinical practice to evaluate joint space narrowing, osteophyte presence, and overall structural joint changes, which are assessed using the Kellgren–Lawrence (KL) grading system [14]. While effective for detecting advanced OA, X‐rays are limited in their ability to detect early and subtle changes in soft tissues, such as cartilage and meniscus, which are critical in identifying early OA development.

MRI, on the other hand, provides a detailed assessment of both bone and soft tissues, allowing for early detection of cartilage loss, meniscal pathology and bone marrow lesions that may precede radiographic changes. This imaging modality offers high sensitivity for early OA changes but is less accessible and more costly than X‐ray. Furthermore, weight‐bearing X‐rays provide critical information on lower limb alignment, a key biomechanical factor influencing OA onset and progression, which MRI does not capture. Due to these limitations, there is a clinical need to optimise the use of X‐rays for early OA diagnosis. Understanding how X‐ray findings relate to MRI‐detected changes can potentially allow clinicians to infer soft tissue conditions from radiographs alone, reducing the need for costly MRI in early stages of disease assessment.

Despite the advantages of MRI in early OA detection, few studies have evaluated the relationship between X‐ray and MRI findings longitudinally and their follow‐up lengths had been relatively short [2, 8, 20]. The availability of longitudinal data over provides an opportunity to understand the temporal correlation between changes in X‐ray and MRI findings, offering insights into the structural progression of OA and potentially identifying early radiographic markers of cartilage or meniscal pathology. Such studies are critical for identifying radiographic indicators that might signal underlying changes detected by MRI, thus bridging the gap between these two imaging modalities and improving early diagnosis and cost‐effectiveness in OA management.

The objective of this study was to evaluate the longitudinal correlation between X‐ray and MRI findings in knee OA, using the Multicenter Osteoarthritis Study (MOST) dataset [10]. By analysing how radiographic changes correspond to MRI‐detected cartilage, meniscal, and bone pathologies over time, this study aims to identify early markers of knee OA progression, thereby enhancing the diagnostic utility of X‐ray and MRI.

## METHODS

The MOST study is a large, prospective, observational study focused on knee OA and comprises data from 3026 individuals aged between 50 and 79 years old. Out of these 6052 knees (two knees from each individual), the knees with both X‐ray and MRI at the same time period (either at baseline or at follow‐ups) were chosen for analysis (3988 knees). To ensure a focused analysis on the more prevalent medial compartment OA, knees with greater lateral JSN than medial JSN were excluded, given the distinct biomechanical and pathological differences between compartments. As a result, a total of 3710 knees were selected for analysis.

Ethical approval for collecting all subject information was provided by the MOST, and informed consent was obtained from all individual participants included in the study. All participants provided informed consent before participating, in accordance with the Helsinki Declaration. Access, download and analyses of the data were performed under the Data Use Agreement of MOST.

**Figure 1:**
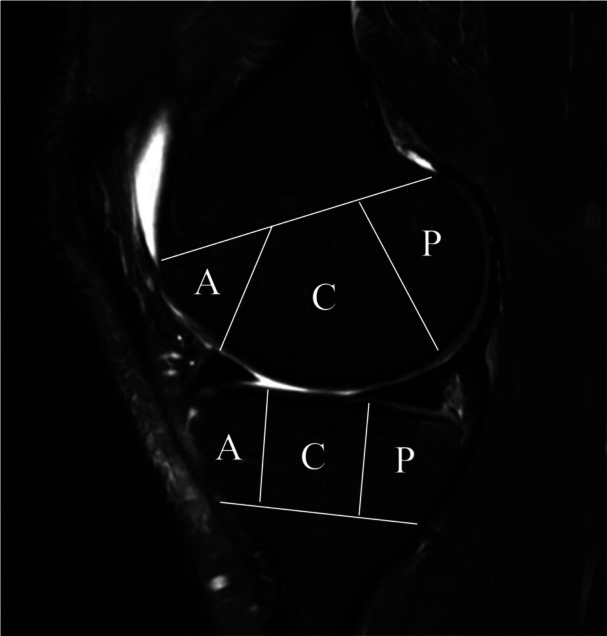
Showing the anterior (A), central (C) and posterior (P) subregions of the medial femoral condyle and medial tibial plateau. Note that anterior subregion of medial femoral condyle is classified into patella‐femoral compartment instead of medial tibio‐femoral compartment.

For X‐rays, JSN grades and osteophyte grades for different locations were obtained, while grades for cartilage, meniscus and bone pathology were assessed for MRIs. The radiographic grades were evaluated according to the Osteoarthritis Research Society International (OARSI) classification system; [1] the MRI grades were evaluated according to the whole‐organ magnetic resonance imaging score (WORMS) classification [19]. Meniscal tears were graded separately for the anterior horn, the body and the posterior horn of the medial and lateral meniscus (grades 0–4). Cartilage morphology (grades 0–6) and bone marrow lesions (grades 0–3) were evaluated in each of the subregions in the medial compartment (Supporting Information S3: Tables S1–S3, respectively). The subregions were divided into anterior, central, posterior in the sagittal plane each for the femur and the tibia (Figure 1). There were five subregions for the medial tibio‐femoral compartment, which was the focus of this particular study, since anterior part of femur was considered as a part of patello‐femoral compartment.

First, MRI findings (cartilage, meniscus and bone pathologies) from different anatomical locations were compared between different radiological grades of knee OA severity (KL grades). MRI findings (cartilage, meniscus and bone pathologies) across KL grades were descriptively compared to illustrate progression patterns without applying inferential statistical tests, as the primary objective was to visualise structural trends rather than test group differences. Additionally, X‐ray findings were compared between different grades of cartilage loss. Finally, using longitudinal data, a mixed linear regression analysis was performed to predict knee OA progression based on MRI factors, including cartilage and meniscal lesions.

For statistical analysis, continuous variables such as age and BMI were summarised using mean and standard deviation (SD), while categorical variables such as sex were reported as frequency and proportion. A *p*‐value cutoff of 0.05 was used to determine statistical significance. Longitudinal data were analysed using mixed linear regression models to predict knee OA progression, incorporating MRI‐based factors such as cartilage and meniscus pathologies as fixed effects. Follow‐up time was included both as a fixed effect, to model the overall effect of time progression, and as a random effect, to account for variability in progression rates between knees. Random effects were also included to adjust for repeated measures within the same knee over time. This approach allowed for modelling individual trajectories of disease progression while accounting for the inherent correlations in longitudinal data.

The demographics of the 3710 knees were as follows: mean age 64.8 ± 8.1 years, mean body mass index (BMI) 29.5 ± 4.7 kg/m², and 60.9% were female (2259 knees). The authors included the same knees from different follow‐ups in calculating demographic findings, as both age and BMI change during the course of follow‐up.

## RESULTS

**Figure 2:**
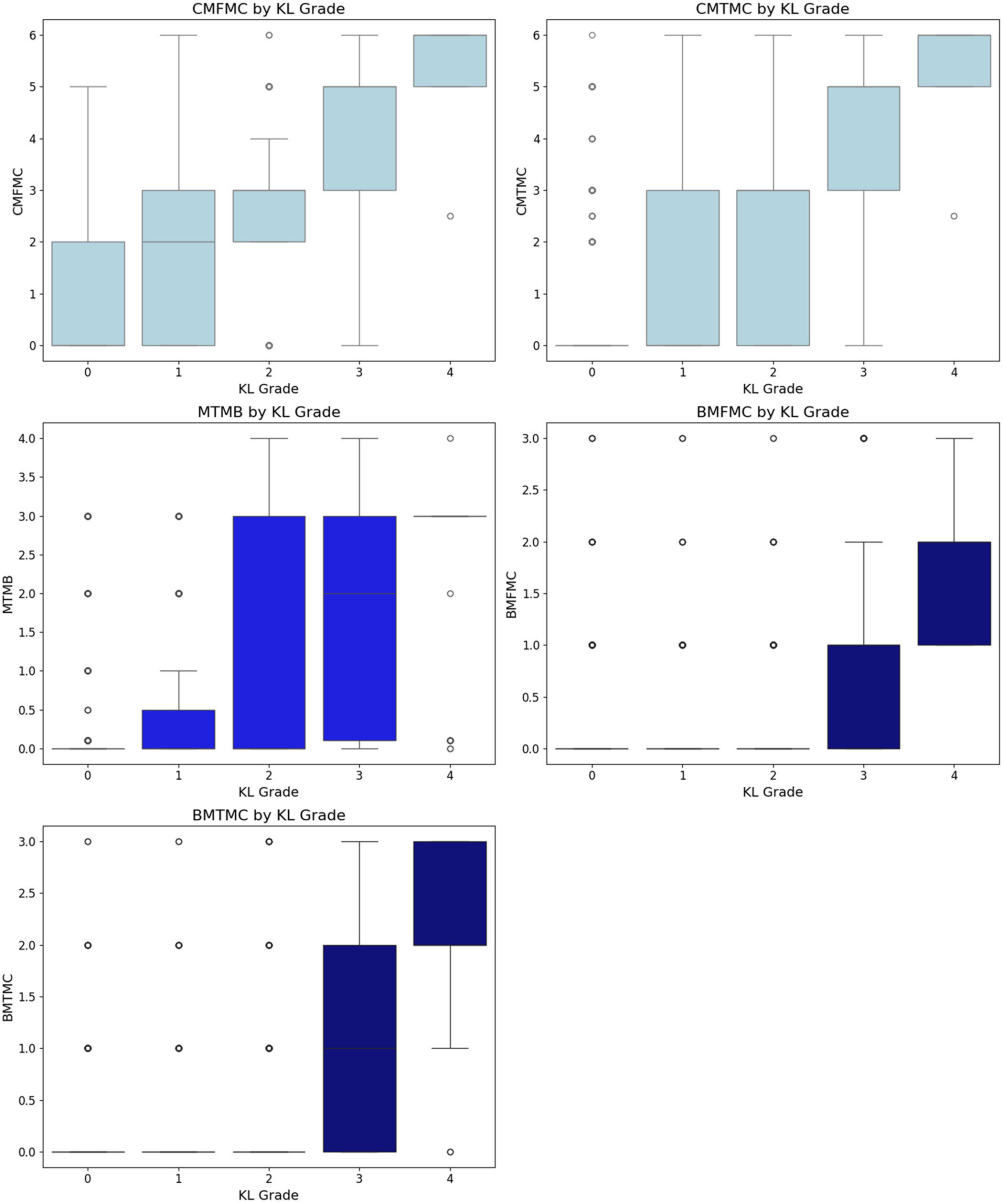
Box‐and‐whisker plots of centro‐medial knee pathologies by KL grade. Note that the lesions start from cartilage (light blue), followed by meniscus (blue) and bone (dark blue). BMFMC, centro‐medial femur bone marrow lesion; BMTMC, centro‐medial tibia bone marrow lesion; CMFMC, centro‐medial femur cartilage morphology; CMTMC, centro‐medial tibia cartilage morphology; KL, Kellgren–Lawrence; MTMB, medial body meniscal tear.

**Figure 3:**
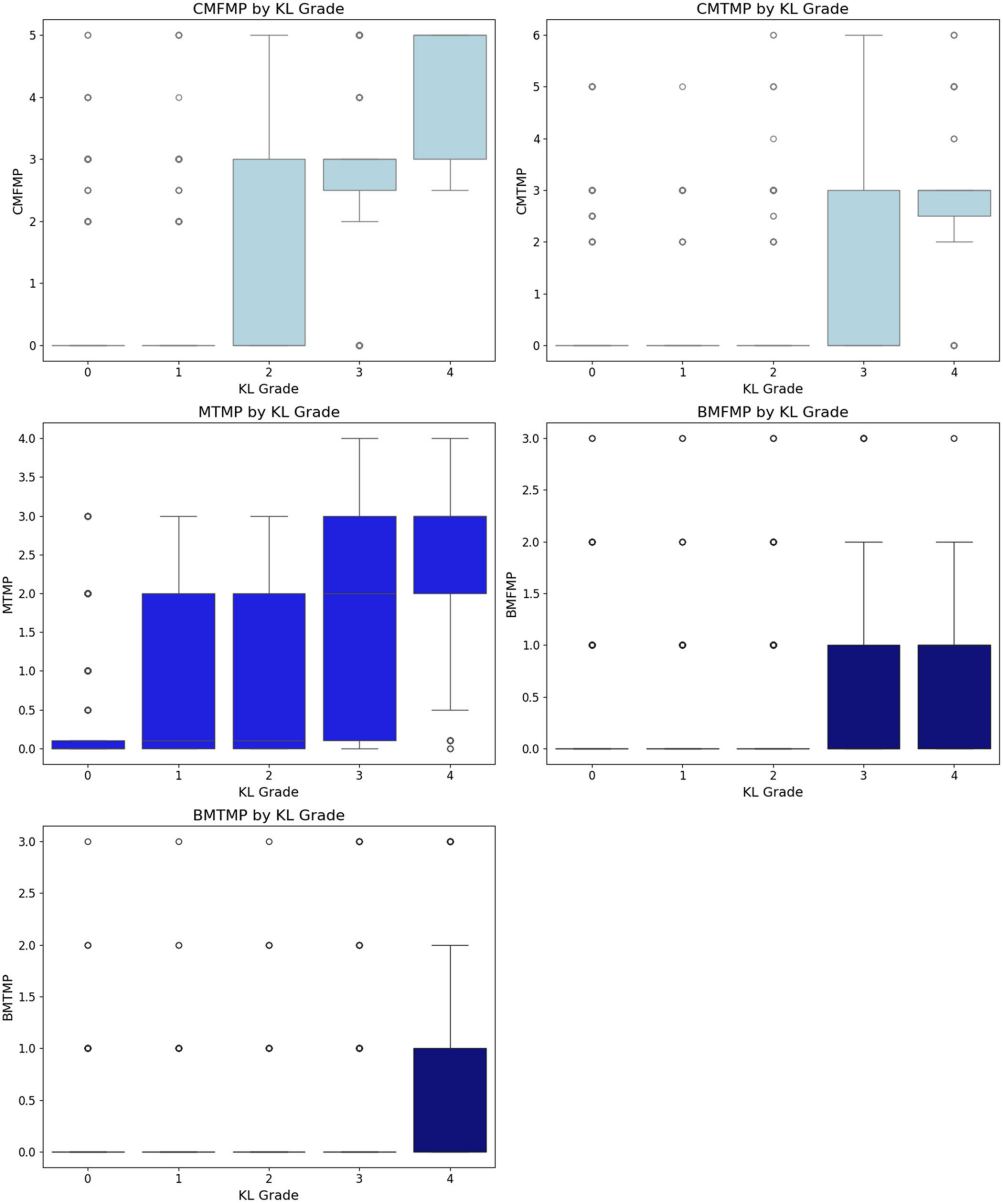
Box‐and‐whisker plots of postero‐medial knee pathologies by KL grade. Note that the lesions start from meniscus (blue), followed by cartilage (light blue) and bone (dark blue). BMFMP, postero‐medial femur bone marrow lesion; BMTMP, postero‐medial tibia bone marrow lesion; CMFMP, postero‐medial femur cartilage morphology; CMTMP, postero‐medial tibia cartilage morphology; KL, Kellgren–Lawrence; MTMP, postero‐medial meniscal tear.

**Figure 4:**
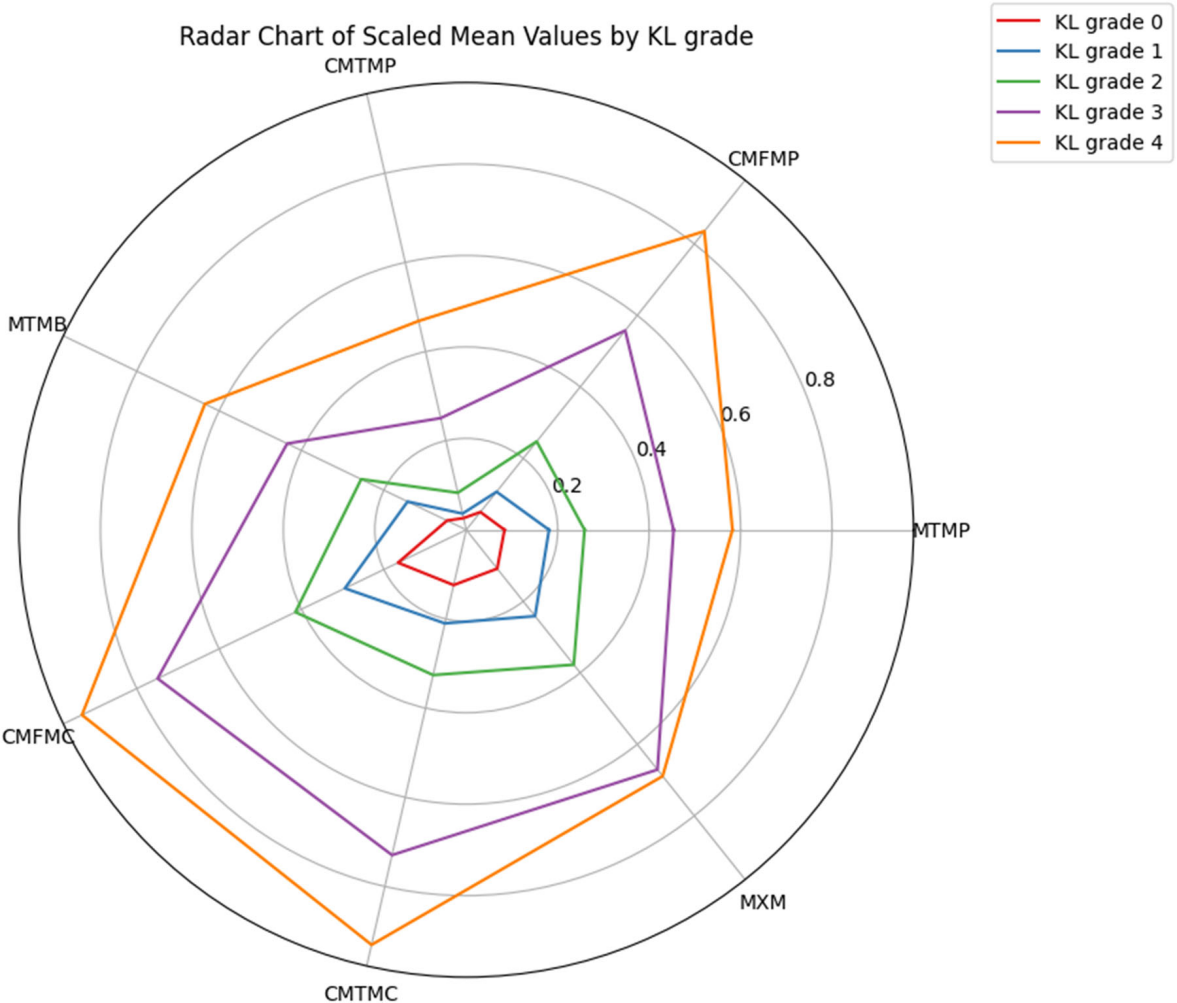
Radar chart of scaled mean values for lesions graded by MRI per osteoarthritis severity. Note that the MRI variables were scaled to 1 for direct comparison between the grades of cartilage, meniscus and bone pathologies. BMFMC, centro‐medial femur bone marrow lesion; BMTMC, centro‐medial tibia bone marrow lesion; CMFMC, centro‐medial femur cartilage morphology; CMTMC, centro‐medial tibia cartilage morphology; KL, Kellgren‐Lawrence; MTMB, medial body meniscal tear.

The location‐specific analysis showed different patterns in MRI over the course of knee OA progression. For the central compartment, cartilage loss developed first followed by meniscal tear and bone oedema (Table 1 and Figure 2). Contrarily for the posterior compartment, meniscal tear developed first followed by cartilage and bone pathology (Table 2 and Figure 3). Combining the MRI findings regardless of lesion location showed that cartilage loss in the central femur tends to occur first even in KL grade 0, preceding observable X‐ray changes. It was followed by medial meniscus extrusion, postero‐medial meniscus tear and then postero‐medial cartilage loss. Figure 4 shows the radar chart of MRI factors (scaled to 1) per knee OA severity. The MRI variables were scaled to 1 for easier comparison between the grades of cartilage, meniscus and bone pathologies.

**Table 1:** Cartilage, meniscus and bone lesions in centro‐medial part of the knee per osteoarthritis severity.

Kellgren–Lawrence grade	CMFMC	CMTMC	MTMB	BMFMC	BMTMC
0	1.0 ± 1.4	0.7 ± 1.4	0.2 ± 0.6	0.1 ± 0.3	0.0 ± 0.3
1	1.8 ± 1.5	1.3 ± 1.7	0.6 ± 1.0	0.1 ± 0.4	0.1 ± 0.4
2	2.5 ± 1.6	2.0 ± 1.9	1.0 ± 1.3	0.2 ± 0.5	0.3 ± 0.6
3	4.5 ± 1.2	4.4 ± 1.4	1.7 ± 1.4	0.7 ± 0.9	1.0 ± 1.0
4	5.6 ± 0.7	5.6 ± 0.7	2.5 ± 1.1	1.8 ± 0.8	2.1 ± 0.8

*Note*: Cartilage, meniscus, bone pathologies are graded according to the whole‐organ magnetic resonance imaging score (WORMS) classification.

Abbreviations: BMFMC, centro‐medial femur bone oedema; BMTMC, centro‐medial tibia bone oedema; CMFMC, centro‐medial femur cartilage morphology; CMTMC, centro‐medial tibia cartilage morphology; MTMB, medial body meniscal tear.

**Table 2:** Cartilage, meniscus and bone lesions in postero‐medial part of the knee per osteoarthritis severity.

Kellgren–Lawrence grade	CMFMP	CMTMP	MTMP	BMFMP	BMTMP
0	0.2 ± 0.8	0.2 ± 0.7	0.3 ± 0.7	0.1 ± 0.3	0.0 ± 0.2
1	0.5 ± 1.1	0.2 ± 0.8	0.7 ± 1.0	0.1 ± 0.4	0.0 ± 0.2
2	1.2 ± 1.6	0.5 ± 1.2	1.0 ± 1.2	0.3 ± 0.6	0.0 ± 0.2
3	2.8 ± 1.6	1.5 ± 1.8	1.8 ± 1.2	0.4 ± 0.7	0.1 ± 0.5
4	4.1 ± 1.0	2.8 ± 1.7	2.3 ± 1.1	1.0 ± 0.8	0.7 ± 1.1

*Note*: Cartilage, meniscus, bone pathologies are graded according to the whole‐organ magnetic resonance imaging score (WORMS) classification.

Abbreviations: BMFMP, postero‐medial femur bone oedema; BMTMP, postero‐medial tibia bone oedema; CMFMP, postero‐medial femur cartilage morphology; CMTMP, postero‐medial tibia cartilage morphology; MTMP, postero‐medial meniscal tear.

The proportions of medial meniscal body and posterior horn tears across KL grades are shown in Figures SA and SB. For KL grades 0–3, significant tears (grade ≥2) were more frequent in the posterior horn than in the meniscal body. Notably, even in KL grade 0, over 10% of knees (11.7%, 219 out of 1876) exhibited significant posterior horn tears. By KL grades 3 and 4, significant tears were observed in more than 60% of cases for both the meniscal body (64.4%, 369 out of 573) and posterior horn (68.6%, 393 out of 573).

**Figure 5:**
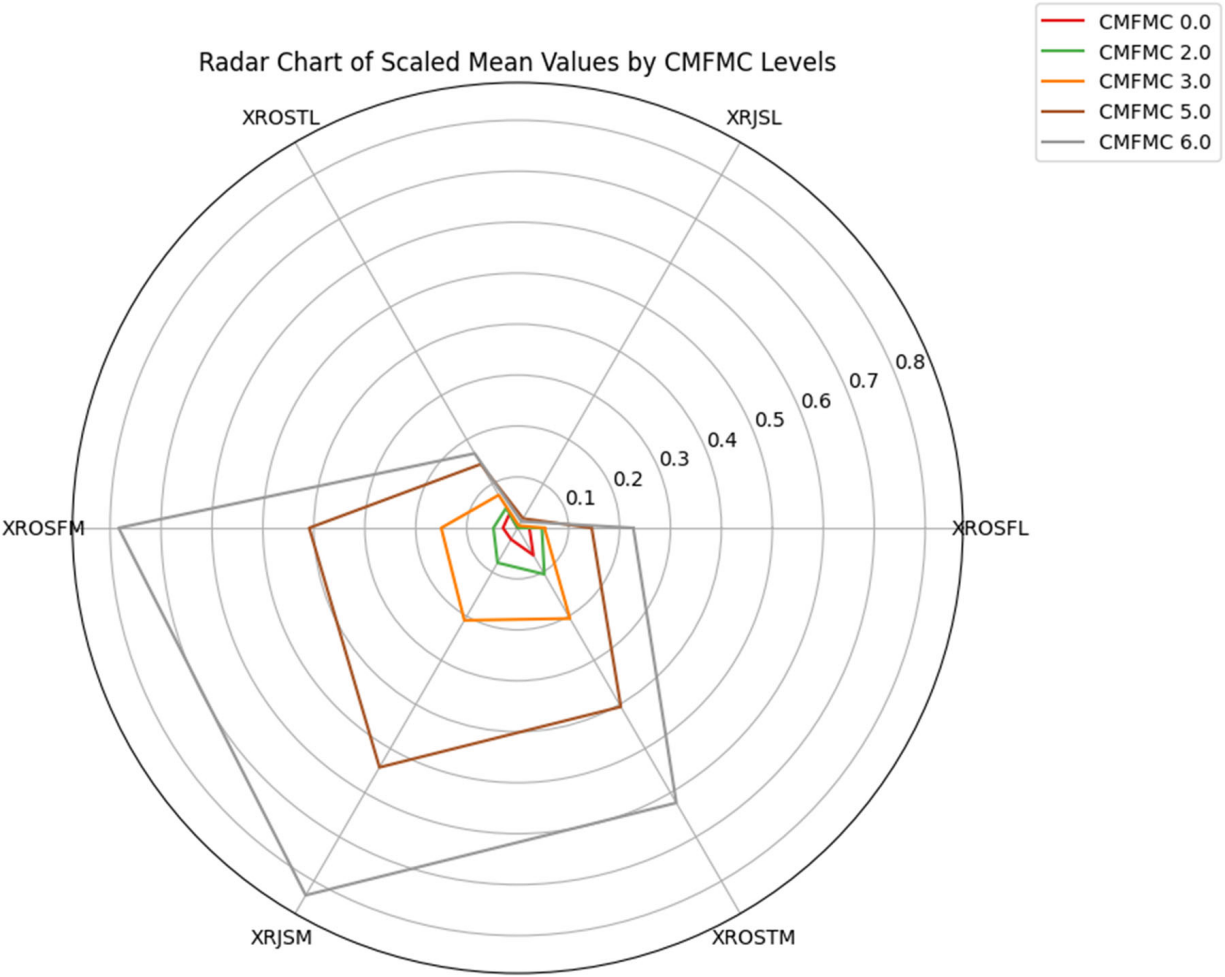
Radar chart of scaled mean values for radiographic grades per cartilage loss in the central femur. The radiographic grades were given according to Osteoarthritis Research Society International classifications. CMFMC, centro‐medial femur cartilage morphology; XRJSL, lateral joint space narrowing grade; XRJSM, medial joint space narrowing; XROSFL, lateral femoral osteophyte grade; XROSFM, medial femoral osteophyte grade; XROSTL, lateral tibial osteophyte grade; XROSTM, medial tibial osteophyte grade.

X‐ray findings were compared between the grades of cartilage loss in the central femur, which was the most sensitive location of cartilage loss during knee OA progression. This analysis showed that tibial osteophytes develop first followed by joint space narrowing and femoral osteophyte formation (Figure 5). The medial JSN and osteophyte grades according to the grades of cartilage loss in the central femur are shown in Table 3.

**Table 3:** Medial joint space narrowing and osteophyte grades per cartilage loss in centro‐medial femur.

CMFMC	JSM	OSTM	OSFM
0	0.1 ± 0.3	0.2 ± 0.4	0.1 ± 0.3
2	0.2 ± 0.5	0.3 ± 0.5	0.1 ± 0.5
3	0.6 ± 0.8	0.7 ± 0.9	0.5 ± 0.8
5	1.6 ± 0.9	1.2 ± 0.8	1.2 ± 1.1
6	2.5 ± 0.5	1.9 ± 0.8	2.4 ± 0.8

Abbreviations: CMFMC, centro‐medial femur cartilage morphology; JSM, medial joint space narrowing grade; OSFM, medial femur osteophyte grad; OSTM, medial tibia osteophyte grade.

Mixed linear regression identified medial meniscal extrusion as the strongest predictor of KL grade progression (coefficient: 0.347, *p* < 0.001, Table 4). Other significant contributors included cartilage morphology in the postero‐medial femur and centro‐medial femur, as well as meniscal tears in the medial body and postero‐medial posterior horn (all *p* < 0.001). Follow‐up time was not a significant predictor (*p* = 0.359), suggesting structural changes rather than time progression primarily drive OA progression. Random effects revealed substantial between‐knee variability (variance: 0.218) with smaller variability in follow‐up effects (variance: 0.038).

**Table 4:** Significant predictors of knee osteoarthritis progression from mixed linear model regression.

	Coefficient	Std.Err.	*z*‐value	*p* value	95% Confidence interval
MXM	0.347	0.022	15.462	**<0.001**	[0.303, 0.391]
CMFMP	0.148	0.011	14.073	**<0.001**	[0.127, 0.169]
CMFMC	0.09	0.01	9.309	**<0.001**	[0.071, 0.109]
MTMB	0.109	0.015	7.477	**<0.001**	[0.081, 0.138]
MTMP	0.107	0.015	7.06	**<0.001**	[0.077, 0.137]
CMTMC	0.047	0.01	4.909	**<0.001**	[0.028, 0.066]
CMTMP	0.018	0.013	1.366	0.172	[−0.008, 0.044]
FU	0.005	0.005	0.917	0.359	[−0.005, 0.015]
Group Var	0.218	0.042			
FU Var	0.038				

Abbreviations: CMFMC, centro‐medial femur cartilage morphology; CMFMP, postero‐medial femur cartilage morphology; CMTMC, centro‐medial tibia cartilage morphology; CMTMP, postero‐medial tibia cartilage morphology; FU Var, variance in the effect of follow‐up time across knees; FU, follow‐up time (months); Group Var, variance between knees; MTMB, medial body meniscal tear; MTMP, postero‐medial meniscal tear; MXM, medial meniscus extrusion.

## DISCUSSION

This study presents a novel longitudinal analysis of X‐ray and MRI findings in medial knee OA, based on a large cohort with 7 years of follow‐up. MRI revealed that structural changes differ by anatomical location, with cartilage loss in the central femur typically occurring first, followed by medial meniscal extrusion, posterior meniscal tears and subsequent cartilage damage in the posterior compartment. In contrast, X‐ray changes generally began with tibial osteophytes, progressing to joint space narrowing and femoral osteophyte formation. However, these radiographic changes appeared later and were less consistent than MRI‐detected alterations. Overall, the findings underscore the compartment‐specific nature of OA progression and highlight MRI's value in detecting early structural changes.

Cartilage loss in the central femur was identified as one of the earliest MRI‐detectable changes, even in radiographically normal knees. This finding is consistent with previous studies that recognise the central femur as a sensitive site for early cartilage degeneration in knee OA [4, 24, 25]. Medial meniscal extrusion emerged as the strongest MRI predictor of KL grade progression (coefficient: 0.347, *p* < 0.001), highlighting its critical role in biomechanical disruption and joint instability [3, 9, 17, 21]. These results emphasise the importance of early MRI assessment to detect structural changes that may not yet appear on radiographs. Early identification of biomechanical disruptions, such as meniscal extrusion, could offer opportunities for targeted interventions aimed at slowing disease progression.

X‐ray findings demonstrated a temporal delay relative to MRI‐detected changes. Tibial osteophytes, commonly the first observable radiographic change, were generally followed by joint space narrowing and femoral osteophytes. However, this sequence was relatively inconsistent when compared to MRI‐detected pathology. The limited sensitivity of X‐rays to early cartilage and meniscal changes underscores the value of MRI in detecting early structural abnormalities [11, 23]. However, tibial osteophytes may still serve as accessible surrogate markers for underlying early knee OA development, particularly in settings with limited access to MRI.

MRI findings revealed distinct patterns of OA progression across compartments. In the central compartment, cartilage loss occurred early, followed by meniscal tears and bone marrow lesions. Conversely, posterior compartment progression began with meniscal changes, such as posterior horn tears and extrusion, before cartilage and bone pathology emerged. These findings are consistent with prior research demonstrating that the detrimental effects of meniscal tears are often localised to the cartilage subregions directly beneath the torn meniscal segments, with posterior meniscal pathology driving cartilage loss specifically in the posterior compartment [6]. In contrast, cartilage loss preceded meniscal body tears in the central region since significant proportion of cartilage in the central region lacks direct contact with the meniscus except for the cases of complete discoid meniscus. The high prevalence of significant posterior horn tears, even in KL grade 0 knees (>10%), underscores meniscal pathology as an early driver of OA progression, often preceding radiographic changes. By KL grades 3 and 4, significant tears were present in over 60% of the body and posterior horn, emphasising the progressive biomechanical disruption that accelerates cartilage loss. Understanding these compartment‐specific patterns is critical for designing tailored interventions, particularly for weight‐bearing compartments most prone to rapid deterioration. It was also interesting to see that contrary to early knee OA, in the late stages of knee OA, the grades of meniscal body tears became even higher than the grades of posterior horn tears (Figures SA and SB).

The mixed linear regression model provided valuable insights into the factors driving OA progression. Medial meniscal extrusion was confirmed as the strongest predictor of KL grade progression, followed by cartilage morphology in the postero‐medial femur (coefficient: 0.148, *p* < 0.001) and the centro‐medial femur (coefficient: 0.090, *p* < 0.001). Notably, follow‐up time was not a significant predictor (coefficient: 0.005, *p* = 0.359), suggesting that structural changes, rather than time alone, primarily drive OA progression. Variance analysis revealed substantial between‐knee variability (Table 4, Group Var: 0.218), indicating significant differences in progression rates across knees. Additionally, the variability in the effect of follow‐up across knees (Table 4, FU Var: 0.038) was modest, suggesting limited differences in time‐related progression between knees. This indicates that while structural changes are the primary drivers of OA progression, some degree of individual variation exists.

The integration of MRI and X‐ray findings provides a robust framework for improving OA diagnosis and management. MRI's ability to detect soft tissue changes complements the accessibility of X‐rays, enabling a synergistic diagnostic approach. Findings such as tibial osteophytes, while not directly reflecting cartilage or meniscal pathology, could serve as cost‐effective surrogate markers for early MRI‐detected changes [5, 13]. The early identification of meniscal extrusion and cartilage loss through MRI provides actionable insights for targeted interventions, potentially delaying or preventing irreversible structural damage.

This study has several limitations that should be considered. First, directly comparing cartilage, meniscus and bone pathologies, even after scaling to a uniform range, may oversimplify their complex interactions. Although WORMS and KL grades are widely used, semiquantitative systems have inherent variability despite established reliability metrics [15]. Second, while the study identified patterns of medial OA progression, there was significant overlap in findings across different KL grades, indicating that the results reflect tendencies rather than absolute distinctions. Third, the study solely focused on medial compartment knee OA and excluded analysis on lateral compartment or patello‐femoral joint. However, isolated lateral compartment knee OAs are relatively few (about 15%) [22] and would show different meniscus and cartilage pathologies and thus were excluded. Although this study focused on medial compartment OA due to its predominance, future research should explore lateral compartment OA independently to validate compartment‐specific progression patterns. Furthermore, potential confounders such as BMI [26] and physical activity [12]—both known to influence OA progression—were not controlled in this study. Higher BMI increases joint loading, while varying physical activity levels may accelerate or mitigate structural changes. These factors could have impacted the observed associations. Future studies should adjust for such variables to better define the independent role of imaging findings in OA progression. Lastly, the use of semi‐quantitative grading systems, such as WORMS and KL grades, does not fully capture qualitative aspects of OA, such as the specific type of meniscal tears (e.g., longitudinal or horizontal). Future studies should address these limitations by incorporating more granular qualitative data, validating findings in larger and more diverse populations, and investigating the clinical utility of meniscal extrusion and other MRI‐detected changes for predicting OA progression. Additionally, developing predictive models that integrate X‐ray and MRI data, while considering cost‐effective imaging strategies, could further optimize early OA management and improve patient outcomes.

Knee OA progression differs between compartments, with cartilage loss being the earliest event in the central compartment and meniscal changes preceding others in the posterior compartment. Tibial osteophytes typically develop before joint space narrowing on X‐ray. Longitudinal analysis highlights meniscal extrusion as the strongest predictor of OA progression, underscoring the utility of MRI in identifying early changes and guiding personalized management strategies.

## AUTHORS CONTRIBUTIONS

**Do Weon Lee**: Collection and assembly of data; statistical analysis; draft of the manuscript. **Ji‐Sahn Kim and Hyuk‐Soo Han**: Statistical analysis; writing and editing. **Du Hyun Ro**: Conception of idea; statistical analysis; editing of manuscript. All authors revised and approved the final manuscript for submission.

## CONFLICT OF INTEREST STATEMENT

The corresponding author, Du Hyun Ro, is the CEO of CONNECTEVE Co., Ltd. However, this had no effect on the results of our research. The remaining authors declare no conflicts of interest.

## ETHICS STATEMENT

This article was prepared using the Multicenter Osteoarthritis Study (MOST) public‐use dataset. Ethical approval for collecting all subject information was provided by the MOST, and informed consent was obtained from all individual participants included in the study. This article does not contain any studies with human participants performed by any of the authors. All participants provided informed consent before participating, in accordance with the Helsinki Declaration. Access, download and analyses of the data were performed under the Data Use Agreement of MOST.

## Supporting information


**Supplementary Figure A.** Proportion of medial meniscal body tear grades (MTMB) across Kellgren‐Lawrence grades.

**Supplementary Figure B.** Proportion of postero‐medial meniscal tear grades (MTMP) across Kellgren‐Lawrence grades.

Supporting information.

## Data Availability

The datasets used in the study are public‐use datasets. The details about acquisition of these dataset are provided in https://most.ucsf.edu/multicenter-osteoarthritis-study-most-public-data-sharing.
