# Supplementary figures and images for: Longitudinal correlation between X‐ray and MRI findings in medial compartment knee osteoarthritis: Insights into early cartilage loss and structural changes
Source: Knee Surg Sports Traumatol Arthrosc. 2025 Aug 31;34(6):2047–56. doi: 10.1002/ksa.70016 (PMC13266911; doi:10.1002/ksa.70016)

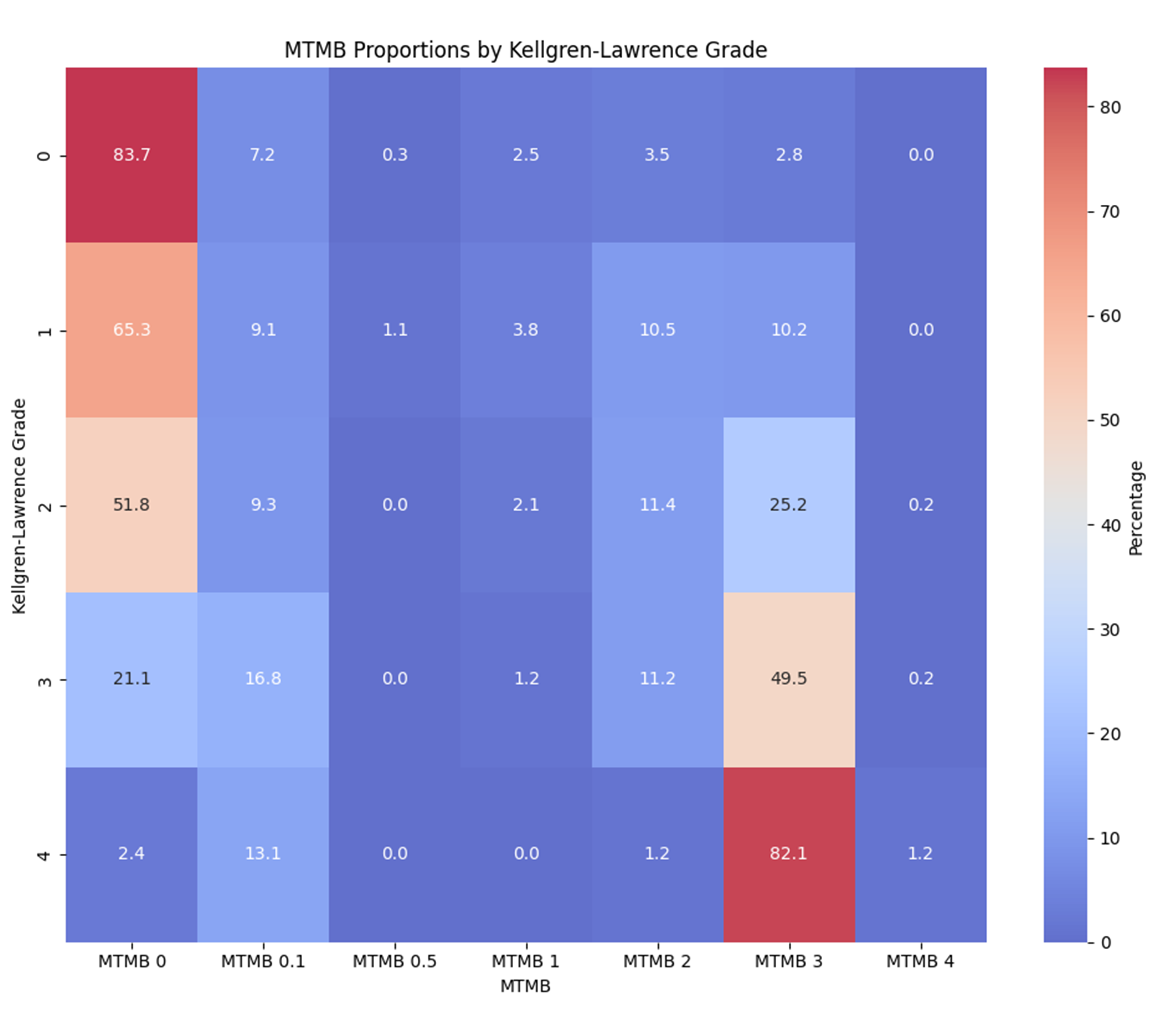

Supplement: Supplementary file 1 — Supplementary Figure A. Proportion of medial meniscal body tear grades (MTMB) across Kellgren‐Lawrence grades. [file KSA-34-2047-s001.tif]

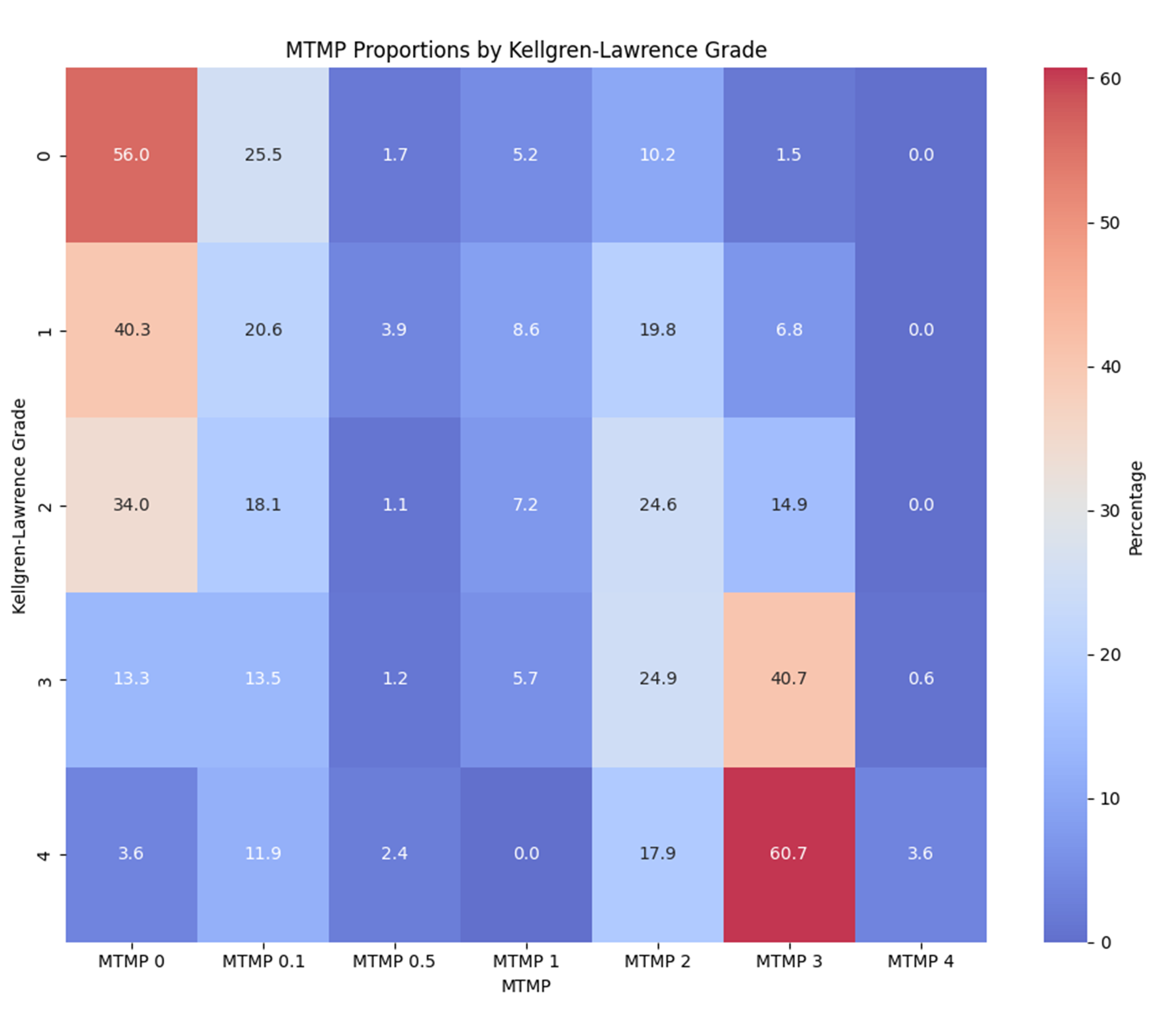

Supplement: Supplementary file 2 — Supplementary Figure B. Proportion of postero‐medial meniscal tear grades (MTMP) across Kellgren‐Lawrence grades. [file KSA-34-2047-s003.tif]
