# Supplementary material for: Longitudinal correlation between X‐ray and MRI findings in medial compartment knee osteoarthritis: Insights into early cartilage loss and structural changes
Source: Knee Surg Sports Traumatol Arthrosc. 2025 Aug 31;34(6):2047–56. doi: 10.1002/ksa.70016 (PMC13266911; doi:10.1002/ksa.70016)
Supplement: Supplementary file 3 — Supporting information. [file KSA-34-2047-s002.docx]

| **Score/Value** | **Description** |
| --- | --- |
| 0 | Intact |
| 1 | Minor radial tear or parrot-beak tear |
| 2 | Non-displaced tear or prior surgical repair |
| 3 | Displaced tear or partial resection |
| 4 | Complete maceration/destruction/resection |

**Supplementary Table 1. Meniscal tear scoring method used in the study.**

| **Score/Value** | **Description** | **Any Cartilage Lesion** | **Full Thickness Loss** |
| --- | --- | --- | --- |
| 0 | Normal thickness | No | No |
| 2 | Partial thickness focal defect | Yes | No |
| 2.5 | Full thickness focal defect | Yes | Yes |
| 3 | Multiple areas of < 1 cm partial-thickness (grade 2) defects intermixed with areas of normal thickness, or a partial thickness defect wider than 1 cm but <75% of the region | Yes | No |
| 4 | Diffuse (>75% of the region) partial-thickness loss | Yes | No |
| 5 | Multiple areas of full-thickness loss (grade 2.5) or a grade 2.5 lesion wider than 1 cm but <75% of the region | Yes | Yes |
| 6 | Diffuse (>75% of the region) full-thickness loss | Yes | Yes |

**Supplementary Table 2. Cartilage morphology scoring method used in the study.**

| **Score/Value** | **Description** |
| --- | --- |
| 0 | None |
| 1 | < 25% of subregion |
| 2 | 25%-50% of subregion |
| 3 | >50% of subregion |

**Supplementary Table 3. Bone marrow lesion scoring method used in the study.**
